# Optogenetic Activation of Astrocytes—Effects on Neuronal Network Function

**DOI:** 10.3390/ijms22179613

**Published:** 2021-09-04

**Authors:** Evgenii Gerasimov, Alexander Erofeev, Anastasia Borodinova, Anastasia Bolshakova, Pavel Balaban, Ilya Bezprozvanny, Olga L. Vlasova

**Affiliations:** 1 Laboratory of Molecular Neurodegeneration, Peter the Great St. Petersburg Polytechnic University, Khlopina St. 11, 194021 St. Petersburg, Russia; alexandr.erofeew@gmail.com (A.E.); bolshakova.av@spbstu.ru (A.B.); Ilya.Bezprozvanny@UTSouthwestern.edu (I.B.); 2 Cellular Neurobiology of Learning Lab, Institute of Higher Nervous Activity and Neurophysiology of the Russian Academy of Science, Butlerova St. 5A, 117485 Moscow, Russia; borodinova.msu@mail.ru (A.B.); pmbalaban@gmail.com (P.B.); 3 Department of Physiology, UT Southwestern Medical Center at Dallas, Dallas, TX 75390, USA

**Keywords:** optogenetics, astrocytes, hippocampal neurons, patch-clamp, channelrhodopsin-2, opto-α1-adrenoreceptor

## Abstract

Optogenetics approach is used widely in neurobiology as it allows control of cellular activity with high spatial and temporal resolution. In most studies, optogenetics is used to control neuronal activity. In the present study optogenetics was used to stimulate astrocytes with the aim to modulate neuronal activity. To achieve this goal, light stimulation was applied to astrocytes expressing a version of ChR2 (ionotropic opsin) or Opto-α1AR (metabotropic opsin). Optimal optogenetic stimulation parameters were determined using patch-clamp recordings of hippocampal pyramidal neurons’ spontaneous activity in brain slices as a readout. It was determined that the greatest increase in the number of spontaneous synaptic currents was observed when astrocytes expressing ChR2(H134R) were activated by 5 s of continuous light. For the astrocytes expressing Opto-α1AR, the greatest response was observed in the pulse stimulation mode (T = 1 s, t = 100 ms). It was also observed that activation of the astrocytic Opto-a1AR but not ChR2 results in an increase of the fEPSP slope in hippocampal neurons. Based on these results, we concluded that Opto-a1AR expressed in hippocampal astrocytes provides an opportunity to modulate the long-term synaptic plasticity optogenetically, and may potentially be used to normalize the synaptic transmission and plasticity defects in a variety of neuropathological conditions, including models of Alzheimer’s disease and other neurodegenerative disorders.

## 1. Introduction

Astrocytes play an integral role in the maintenance and regulation of neural networks in the brain. They are able to influence neuronal activity by regulating the extracellular concentration of potassium ions, as well as neurotransmitters, due to the expression on their membrane of a large number of transporters of electrogenic transmitters such as glutamate [1,2], gamma-aminobutyric acid [3,4], and glycine [5,6]. By releasing gliotransmitters, astrocytes act on neuronal receptors, modulating neuronal excitability, synaptic transmission, and synaptic plasticity. Astrocytes do not generate action potentials in response to a stimulus, but respond with intracellular increase of [Ca^2+^] [7]. When Ca^2+^ waves propagate in astrocyte cytoplasm, serine, cytokines, and lactate are released, which can modulate activity of neighboring neurons [8]. The ability of astrocytes to release glutamate allows regulation of the function of NMDA receptors, thereby controlling the excitation of neuronal network [9]. 

Astrocytes are also an irreplaceable part of the tripartite synapse, which involves coordinated activity of pre- and postsynaptic membrane and astrocytes [10,11]. Their activity is closely related to synaptic potency [12] and is controlled via several types of metabotropic receptors linked to calcium levels. Release of gliotransmitters from astrocytes occurs in both calcium-dependent and calcium-independent ways. The ability of astrocytes to release gliotransmitters in a millisecond time scale is critical for their role in integration of information in neuronal networks [13].

Astrocytes are closely related to the pathogenesis and pathological processes in the brain and, in particular, in the context of Alzheimer’s disease (AD) [14,15,16,17,18,19]. AD [20] is characterized by a progressive memory loss and cognitive dysfunctions, accumulation of a significant number of Aβ-amyloid plaques [21], abnormal neuronal calcium homeostasis [22], and accumulation of neurofibrillary tangles [23]. Shift in excitation and inhibition balance in neuronal network is often considered one of the causes of AD pathology [24,25]. It has been proposed that regulation of neuronal network activity in AD by stimulation of astrocytes may lead to beneficial effects by stabilizing activity of the network [26]. 

Optogenetic techniques allow selective and precise regulation of cellular activity [27,28]. In the present study, the optogenetic approach was used to stimulate activity of astrocytes. For optogenetic activation of astrocytes, two different opsins were used: ChR2 [29] that acts as an ion channel and metabotropic opsin Opto-a1AR [30], stimulation of which leads to activation of IP3 receptor and elevation of cytosolic calcium concentration. In this study, we performed comparison of effects on neuronal function resulting from activation of astrocytes by these two optogenetic tools. Obtained results are useful for future experimental evaluation of astrocyte activation in the context of AD and other neurodegenerative disease models. 

## 2. Results

### 2.1. Specificity of Expression of AAV2/5 GfaABC1D_ChR2(H134R)-mCherry and AAV2/5 GfaABC1D_Opto-a1AR-EYFP

**Figure 1:**
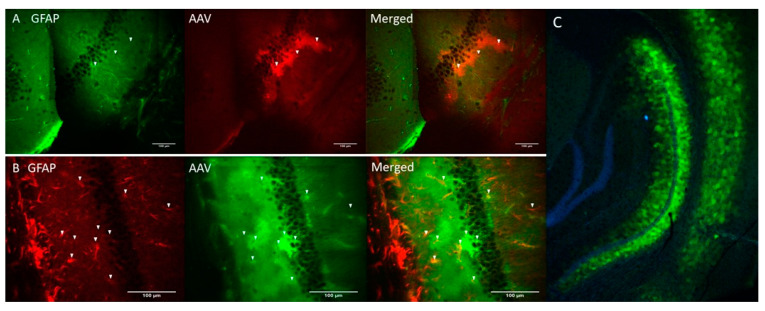
Astrocyte-specific expression of ChR2(H134R)-mCherry and Opto-a1AR-EYFP constructs. (**A**) Confocal images of fixed slices of mouse brain tissue three weeks after unilateral administration of AAV2/5 GfaABC1D_ChR2(H134R)–mCherry (red). GFAP staining (green) was used to label astrocytes. Scale bar is 100 µm. (**B**) Confocal images of fixed slices of mouse brain tissue three weeks after unilateral administration of AAV2/5 GfaABC1D_Opto-a1AR_EYFP (green). GFAP staining (red) was used to label astrocytes. Scale bar is 100 µm. (**C**) Confocal image of fixed slice of mouse brain tissue three weeks after unilateral an administration of AAV2/5 GfaABC1D_Opto-a1AR_EYFP (green), nuclei are labeled with DAPI (blue), 4× magnification.

Genetic constructs of ChR2(H134R) [29] and Opto-a1AR [30] were obtained and packaged into AAV2/5 adeno-associated viruses (AAV) under control of astrocyte-specific GfaABC1D promoter (see Materials and Methods). AAV-ChR2(H134R)-mCherry and AAV-Opto-a1AR-EYFP viruses were stereotaxically injected into hippocampal region of the mice (C57BL/6J strain) and immunohistochemical experiments were performed 3 weeks after injection to verify specificity of transgene expression. Obtained results confirmed co-localization of ChR2-mCherry and astrocytic marker glial fibrillary acid protein (GFAP) (Figure 1A). Similar results were obtained with Opto-a1AR-EYFP (Figure 1B). Obtained results confirmed astrocyte-specific expression of both constructs, in agreement with known specificity of GfaABC1D promoter [31]. Astrocyte-specific expression of Opto-a1AR-EYFP construct was further confirmed by high resolution confocal imaging of mouse hippocampal slices combined with nuclei labeling by DAPI staining (Figure 1C).

### 2.2. Activation of Astrocytes Expressing ChR2(H134R) or Opto-α1AR Leads to Enhancement of Pyramidal Neuron’s Activity 

**Figure 2:**
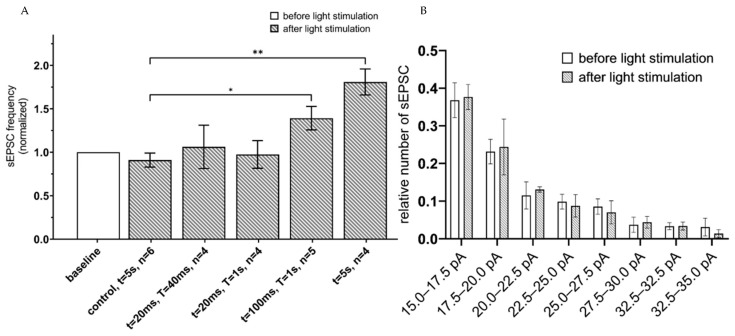
sEPSC changes in response to astrocyte activation using ChR2(H134R)–mCherry. (**A**) Values of the normalized frequencies of sEPSC of hippocampal neurons in the CA1 region after light activation (t = 20 ms, T = 20 ms; t = 20 ms, T = 1 s; t = 100 ms, T = 1 s; continuous light t = 5 s) of astrocytes expressing ChR2(H134R)—mCherry. The data are presented as the mean ± SEM, **: *p* < 0.01, *: *p* < 0.05. (**B**) distribution of sEPSC amplitudes before and after optogenetic (t = 5 s) activation of astrocytes expressing ChR2(H134R)—mCherry on the membrane. The data are presented as the mean ± SEM, *n* = 4.

To evaluate functional effects of astrocyte activation on neuronal function, spontaneous excitatory postsynaptic currents (sEPSC) were recorded in a whole-cell configuration from hippocampal slices 3 weeks after unilateral injection of ChR2(H134R)-mCherry and Opto-a1AR-EYFP expressing viral constructs. Intracellular recording was performed in the presence of 1 µM tetrodotoxin (TTX) in the pipette solution to prevent neurons from action potential generation in normal ACSF as extracellular media. Patch-clamp recordings were performed from neurons located at approximate depth 100 µm from the surface of a slice, where all processes of neurons and astrocytes were undamaged. To determine optimal parameters of optogenetic activation of astrocytes that expressed ChR2(H134R)-mCherry, different protocols of light administration (λ = 473 nm) were applied with variable intervals of stimulation (T) and duration of the light pulses (t). The conditions that were tested: (t = 20 ms, T = 20 ms); (t = 20 ms, T = 1 s); and (t = 100 ms, T = 1 s) and continuous light (t = 5 s). In each experiment, frequency of sEPSC following light stimulation was normalized to the frequency of sEPSC in the same neuron prior to light stimulation. Highest increase in the frequency of sEPSC currents was observed in a group with 5 s of continuous light stimulation (mean increase to 1.81 ± 0.15, *n* = 4) and in a t = 100 ms, T = 1 s group (the mean increased to 1.39 ± 0.14, *n* = 5) (Figure 2A). We further found that optogenetic stimulation of hippocampal astrocytes did not affect amplitudes of spontaneous currents in neurons, with no difference before and after the optogenetic stimulation in distribution of EPSCs amplitudes at all stimulation protocols (*n* = 4, *p* > 0.05, Mann–Whitney U test) (Figure 2B). To rule out potential effects of phototoxic damage and/or heating of a slice by pulses of light, control recordings were performed from hippocampal slices of non-injected hemisphere of the same mice. In these control experiments, we discovered that continuous light stimulation with t = 5 s led to non-significant changes in sEPSC frequency compared to baseline level value (mean 0.91 ± 0.08, *n* = 6, *p* > 0.01, Mann–Whitney U test) (Figure 2A). Increase of sEPSC’s frequency suggests that in ChR2(H134R) expressing astrocytes the optogenetic activation may lead to activation of spontaneous glutamate release in pyramidal neuron synapses, as has been previously shown for visual cortex neurons [32]. 

**Figure 3:**
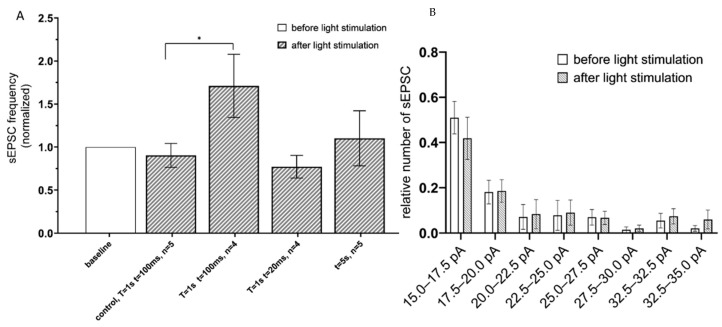
sEPSC changes in response to astrocyte activation using Opto-a1AR-EYFP. (**A**) Values of the normalized frequencies of sEPSC in the CA1 hippocampal neurons after light activation (t = 20 ms, T = 20 ms; t = 20 ms, T = 1 s; t = 100 ms, T = 1 s; continuously t = 5 s) of astrocytes expressing Opto-a1AR-EYFP. The data are presented as the mean ± SEM, *: *p* < 0.05. (**B**) Distribution of sEPSC amplitudes before and after optogenetic activation of astrocytes expressing Opto-a1AR-EYFP on the membrane. The data are presented as an average ± SEM.

To determine an effect of Gq-coupled opsin activation, the hippocampal CA1 astrocytes expressing the Opto-α1AR-EYFP were optogenetically stimulated, and sEPSC recorded using the same approach as described above for ChR2(H134R)-mCherry. To identify optimal parameters of stimulation, the following optogenetic protocols were applied: (t = 20 ms, T = 1 s); (t = 100 ms, T = 1 s) and continuous light (t = 5 s). The greatest increase in the frequency of currents was observed in the group with (t = 100 ms, T = 1 s) (the mean increased to 1.71 ± 0.36, *n* = 4) (Figure 3A). No difference in sEPSC frequency was observed in control experiments in slices from non-injected hemisphere under the same stimulation conditions (t = 100 ms, T = 1 s) (Figure 3A). Similar to experiments with ChR2(H134R)-mCherry, optogenetic stimulation of Opto-a1AR-EYFP had no significant effect on distribution of sEPSC amplitudes (Figure 3B). 

The obtained results suggest that optogenetic stimulation of astrocytes expressing ChR2(H134R)-mCherry or Opto-α1AR-EYFP constructs elicited gliotransmitters release that increased frequency of spontaneous neuronal activity of hippocampal pyramidal neurons but had no effect on the amplitudes of sEPSC currents. 

### 2.3. Activation of Astrocytes Expressing ChR2(H134R)-mCherry Had No Effect on the Field Excitatory Postsynaptic Potentials 

**Figure 4:**
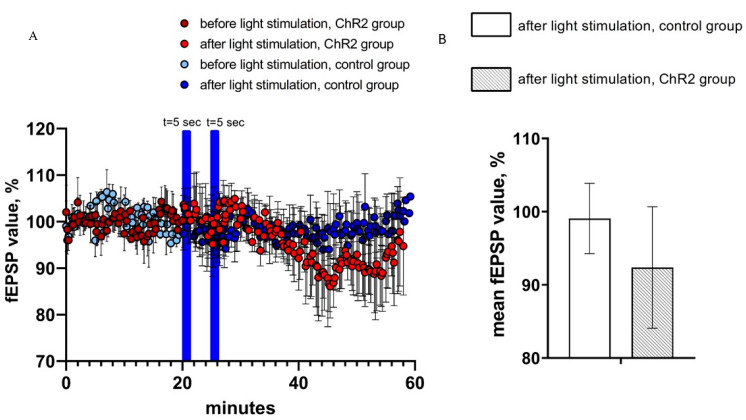
fEPSP changes in response to astrocyte activation using ChR2(H134R)–mCherry. (**A**) the normalized values of fEPSP slope (%) before and after light stimulation of astrocytes in the experimental (ChR2 expressing) and control groups. In the figure, the blue bars show the time of continuous light stimulation. (**B**) Average value of the normalized fEPSP value (%) after light stimulation (2 pulses t = 5 s) in the control and experimental groups, *n* = 4 for control measurements, *n* = 3 in the experimental group. The data are presented as an average ± SEM.

In the next series of experiments, the effects of optogenetic activation of astrocytes on CA1 hippocampal field excitatory postsynaptic potentials (fEPSPs) were evaluated. To achieve fEPSP recordings, hippocampal Shaffer collaterals were stimulated via twisted bipolar electrodes before and after optogenetic activation of astrocytes expressing ChR2(H134R)-mCherry by 5 s of continuous light stimulation. As in previous experiments, hippocampal slices from non-injected hemispheres were used as a control. On average, the value of normalized fEPSP slope in the last 5 min prior to light stimulation was equal to 92.4 ± 8.3 (*n* = 3) in experimental group and 99.1 ± 4.8 (*n* = 4) in control group (Figure 4A). We discovered that two light pulses of 5 s light duration had no significant effect on the average value of the normalized fEPSP slope (%) in both control and experimental groups (ChR2-group (*n* = 3) vs. control group (*n* = 4), *p* > 0.05, Mann–Whitney U test) (Figure 4B). We further noticed that fEPSPs recorded in the experimental (ChR2) group appears to be reduced after light stimulation when compared to the control group (Figure 4A), but the difference has not reached a level of statistical significance (Figure 4B). 

### 2.4. Activation of Opto-α1AR-EYFP-Expressing Astrocytes Leads to Increase of Field Excitatory Postsynaptic Potential 

**Figure 5:**
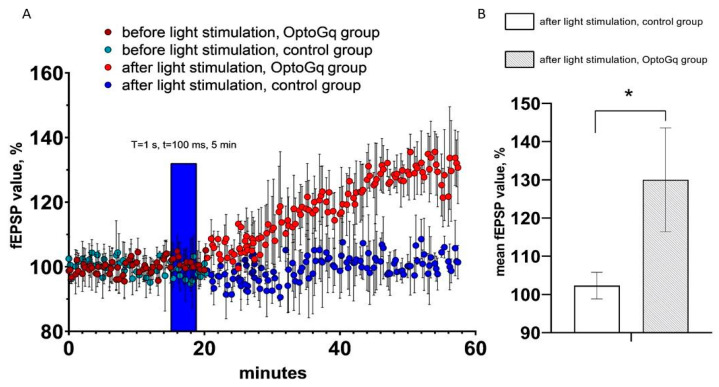
fEPSP changes in response to astrocyte activation using Opto-a1AR-EYFP. (**A**) Normalized value of slope (%) before and after light stimulation of astrocytes in the control and experimental (Opto-a1AR expressing) groups of mice. In the figure, the blue bar marks the timing of light stimulation. (**B**) Average value of the normalized slope value after light stimulation (T = 1s, t = 100ms) in the control and experimental groups mice, *n* = 3 for control measurements, *n* = 3 in the experimental group. The data are presented as an average ± SEM, *: *p* < 0.05.

By using the same approach as for ChR2(H134R)-mCherry, we evaluated changes in hippocampal fEPSP in response to optogenetic activation of astrocytes transduced with Opto-α1AR-EYFP viral construct. T = 1 s and t = 100 ms were chosen as the light stimulation protocol in these experiments, as this protocol resulted in the highest increase in sEPSC (Figure 3A). As in previous experiments, hippocampal slices from non-injected hemispheres were used as a control. In these experiments, the average normalized slope (%) in last 5 min of recordings in experimental (Opto-α1AR) group was 130.0 ± 13.6 (*n* = 3), while in control group it was 102.3 ± 3.5 (*n* = 3). Obtained results demonstrated that light stimulation of astrocytes expressing Opto-a1AR-EYFP leads to significant potentiation of fEPSP (Figure 5A,B). From these results we concluded that Opto-α1AR-EYFP-mediated activation of astrocytes is able to potentiate the hippocampal synaptic transmission significantly, resulting in enhanced fEPSP. 

## 3. Discussion

In this study, the parameters of optogenetic activation of astrocytes that lead to enhancement of neuronal activity were defined. It was determined that the mode of 5 s continuous light stimulation of ChR2(H134R), expressed in astrocytes, provides the highest increase in spontaneous neuronal activity. Impulse mode with T = 1 s, t = 100 ms parameters had maximal effect on spontaneous neuronal activity when Opto-a1AR was used to activate astrocytes. Furthermore, our results and the data from the literature [33] suggest that stimulation of Opto-a1AR expressed in hippocampal astrocytes has a potential for enhancing the long-term synaptic plasticity in mice. In contrast, no significant effect on fEPSP was observed in experiments with astrocytes expressing ChR2(H134R). Possible mechanism responsible for synaptic plasticity changes following activation of Opto-a1AR in astrocytes may be related to secretion of glutamate and d-serine, both of which can potentiate synaptic plasticity [34]. 

Ionotropic opsins are well studied in different types of cells in the nervous system. Effects of ChR2 activation in astrocytes have been previously described [35,36,37,38]. It was demonstrated that activation of ChR2 in astrocytes triggers a release of glutamate and increase in the frequency of spontaneous excitatory postsynaptic currents in pyramidal neurons [38], in agreement with our findings (Figure 2). Activation of ChR2 in astrocytes can also have positive influence on interneurons’ excitability and negative influence on pyramidal neuron activity, what reduces their action potential frequency. It is possible that different and even opposite effects of astrocytic ChR2 activation may be related to various regimes of illumination [36,38] and to different neuronal activity patterns [39].

It was previously shown that chemogenetic activation of hM3Dq-expressing astrocytes by CNO in the hippocampal CA1 neurons leads to increase in mEPSC and synaptic potentiation [33]. Optogenetic activation of Gq-signaling in astrocytes in our experiments did not result in such strong and fast changes in synaptic plasticity. As an explanation, it can be proposed that due to quite low membrane resistance in astrocytes (due to the presence of gap-junctions [40]) more time is needed for more gentle activation by light in comparison with DREADD (Designer Receptors Exclusively Activated by Designer Drugs). In another study, activation of G-protein coupled receptors in astrocytes was performed by expression of melanopsin. In this case, the optogenetic low-frequency stimulation led to a robust EPSC potentiation that persisted after 30 min of recording [37]. Moreover, authors showed that expression of melanopsin in hippocampal astrocytes caused an elevation of IP3-dependent Ca^2+^ signal in their fine processes. It was also reported that light stimulation of astrocytes transfected with Opto-a1AR resulted in a raise of the Ca^2+^ concentration [30]. Increase in astrocytic Ca^2+^ concentration and release of glutamate is most likely an explanation for increased sEPSC frequency following activation of Opto-a1AR in our experiments.

The potentiation of field potentials during optogenetic activation of astrocytes expressing Opto-a1AR is observed in connection with the release of not only glutamate, but also gliotransmitters—for example, d-serine, which is necessary for the formation of long-term changes in plasticity. The release of d-serine occurs through Ca^2+^—and SNARE-dependent exocytosis along with that occurring through alternative non-exocytotic pathways [41]. According to the literature data [42], activation of the metabotropic opsin Opto-a1AR leads to a significant increase in the intracellular concentration of Ca^2+^, which can increase the excretion of serine from astrocytes into the intracellular space. This increase is associated with the activation of intracellular calcium depots. This gliotransmitter may not be released by astrocytes when they activate ChR2, since there is not such a strong increase in the concentration of intra-astrocyte calcium, which is mediated both by its flow through the plasma membrane and by the involvement of intracellular calcium stores. It might be the reason for the lack of a field potential potentiation effect after the activation of ChR2. Obtained results suggest a possibility for regulating the neuronal networks functioning by using light stimulation of Opto-a1AR opsin expressed in astrocytes with parameters defined in this study. Potentially, this approach can be used for correcting the neuronal network dysfunction and improving the synaptic plasticity in a variety of neuropathological conditions, including models of Alzheimer’s disease and other neurodegenerative disorders. Importantly, stimulation of astroglia can also convert it into reactive glia with cytotoxic activity, which can provoke the death of neurons and exacerbate neuroinflammation. In vivo experiments with optogenetic stimulation of astrocytes in mouse models of AD and other disorders are needed to evaluate validity of stimulation parameters defined in our study with brain slices and to refine the proposed experimental approach in order to avoid potential negative effects of astrocyte stimulation on brain function.

## 4. Materials and Methods

### 4.1. Animals

The breeding colony of C57BL/6J mice obtained from the Jackson Laboratory was established and maintained in a vivarium with 4–5 mice per cage and a 12 h light/dark cycle in the animal facility, and were used for the fEPSP experiments. This line was taken because the 5xFAD mice with Alzheimer’s disease genetic model were made using this genetic line, and future experiments are planned to be conducted on them. Albino outbred mice (Rappolovo farm, Leningradsky District, Russia) were used for patch-clamp experiments on acute hippocampal slices. All procedures were approved by principles of the European convention (Strasburg, 1986) and the Declaration of International Medical Association regarding the humane treatment of animals (Helsinki, 1996) and approved by the Bioethics Committee of the Peter the Great St. Petersburg Polytechnic University at St. Petersburg, Russia (Ethical permit number 2-n-b from 25 January 2021).

### 4.2. Plasmids and Production of Viral Constructs

For selective expression of channel rhodopsin in astrocytes we used plasmid AAV pZac2.1 GfaABC1D_ChR2(H134R)-mCherry (Addgene, #112496) that contains short version of astrocyte-specific GFAP promoter GfaABC1D [43]. Opto-a1AR encoding plasmid was generated on the basis of pZac2.1 as follows: Opto-a1AR-EYFP fragment was amplified by PCR using pcDNA3.1/opto-a1AR-EYFP plasmid (Addgene #20947) as a template, and then cloned into pZac2.1 plasmid using NheI/XbaI restriction enzymes to replace the ChR2(H134R)-mCherry fragment. Resulting plasmid AAV GfaABC1D_Opto-a1AR-EYFP was verified by sequencing. The payloads were packaged using commercially available plasmid with AAV5 serotype (Addgene, #104964) to generate recombinant AAV2/5 viruses.

Production of viral particles was carried out according to the standard protocols used for AAV preparation. Briefly, HEK293T cells were plated on polylysine-coated Petri dishes and grown in a DMEM medium supplemented with 10% of FBS until a density of 70–80% was reached. Next, HEK293T cell were subjected to the triple-plasmid transfection using PEI reagent. At the day 3 post-transfection, media and cells were collected and processed separately. Cells were harvested and subjected to freeze/thaw cycles in liquid nitrogen. Supernatants were treated with polyethylene glycol (PEG, Sigma-Aldrich, St. Louis, MO, USA) 8000, the PEG-precipitated AAVs were collected by centrifugation. Viral particles, extracted from cells and supernatant, were combined and treated with benzonase nuclease to destroy any unpacked DNA. Then AAV particles were purified by iodixanol gradient ultracentrifugation. The required fraction, enriched with viral particles, was collected, filtered, and transferred to the Amicon Ultra-15 centrifugal filter unit for buffer exchange and concentration of virus suspension to the final volume of 110–130 µL. Virus titer was then determined by quantitative PCR using primer pair targeting AAV2 ITR sequence in the construct (Forward: GGAACCCCTAGTGATGGAGTT; Reverse: CGGCCTCAGTGAGCGA). To remove any extra-viral DNA before qPCR measurements, virus aliquots were treated with DNase I. Resulting virus titer was equal to 9.1 × 10^14^ vg/mL for AAV2/5 GfaABC1D_ChR2(H134R)_mCherry, and 2.93×10^14^ vg/mL for AAV2/5 GfaABC1D_opto-a1AR_EYFP.

### 4.3. Viral Constructs Delivery via Stereotaxic Surgery

For viral constructs (AAV2/5 pZac2.1 GfaABC1D_ChR2(H134R)_mCherry and AAV2/5 GfaABC1D_Opto-a1AR_EYFP) delivery to the hippocampus, mice aged ~2 months and weighing 24–26 g were used. Injections of viral constructs were performed using a stereotaxic device (68001, RWD Life Science, Guangdong, China), a syringe with a thin needle (84,853, 7758-02, Hamilton, Reno, NV, USA), as well as a heated mat and a temperature controller (69,002, RWD Life Science, Guangdong, China). Surgery was carried out under anesthesia of the animals by anesthetizing 1.5–2.5% with a gas mixture of isoflurane. After a control check of the depth of anesthesia in the animal, the viral constructs were administered according to the standard protocol [44] at the following coordinates: AP−2.1, DV−1.8, ML+2.4, with a volume of 1.5 µL at a rate of 0.1 µL/min. 

### 4.4. Immunohistochemistry 

To test the specificity of injected viruses, three weeks after injection the immunohistochemical staining of mouse brain tissue sections was performed according to the standard protocol [45]. For this purpose, mice were anesthetized by intraperitoneal injection of urethane solution (250 mg/mL in 0.9% NaCl, Sigma-Aldrich, St. Louis, MO, USA). Then transcardial perfusion was performed with PBS followed by standard 1.5% paraformaldehyde solution (30–50 mL, PFA, Sigma-Aldrich, USA). The brain was removed and placed in a 4% PFA solution for post-fixation for 1 week at +4 °C. Fixed brain slices with a thickness of 20–50 microns were obtained using a microtome (5100 MZ, Campden Instruments, United Kingdom) in a PBS solution and stored in a 24-well plate filled with 0.5% PFA. 

Permeabilization of fixed tissue was performed using 0.1% Triton X-100 in PBS, then slices were placed in a blocking buffer (5% BSA in PBS) for 6 h at room temperature. After 6 h, the slices were placed in a solution with primary antibodies (2.5% BSA in PBS-1 mL, 0.2% Tween20-20 µL, antibodies-1 µL, dilution 1/1000) for 8 h on a shaker at +4 °С. Primary antibodies—Anti-GFAP (644701, Biolegend, San Diego, CA, USA)—were used for astrocyte staining. After 8 h primary antibodies were washed and slices were incubated with secondary antibodies (Goat anti-Mouse IgG (H + L) Cross-Adsorbed Secondary Antibody, Alexa Fluor 488, Invitrogen A-11001, dilution 1/2000) for immunostaining of astrocytes expressing ChR2-mCherry, and for experimental group of astrocytes expressing Opto-a1AR-EYFP, the following secondary antibodies were used (Goat anti-Mouse IgG (H + L) Cross-Adsorbed Ready probes Secondary Antibody, Alexa Fluor 594, Invitrogen R37121, dilution 1/1000). Within 8 h after the addition of secondary antibodies, the sections were incubated on a shaker at a low speed at +4 °C. 

### 4.5. Slice Electrophysiology 

Transcardial perfusion was performed with saturated carbogen (95% O_2_ / 5% CO_2_) modified 0–2 °C solution of ACSF (92 mM NMDG, 2.5 mM KCl, 1.25 mM NaH_2_PO_4_, 30 mM NaHCO_3_, 20 mM HEPES, 25 mM d-glucose, 2 mM Thiourea, 5 mM Na-ascorbate, 3 mM Na-pyruvate, 0.5 mM CaCl_2_, 10 mM MgSO_4_) and decapitation was performed. Horizontal slices of the brain with a thickness of 350 microns for the patch-clamp and 400 microns for recording excitatory field potentials were made using a microtome (Leica VT1200S (Leica Biosystems Division of Leica Microsystems Inc., Buffalo Grove, IL, USA)) from 3-month-old mice and maintained in a 0–2 °C solution of NMDG-ACSF saturated with carbogen. The hippocampus was isolated from each slice and incubated in modified ACSF solution (92 mM NaCl (Sigma-Aldrich, St. Louis, MO, USA), 2.5 mM KCl (Sigma-Aldrich, St. Louis, MO, USA), 1.25 NaH_2_PO_4_ (Sigma-Aldrich, St. Louis, MO, USA), 30 mM NaHCO_3_ (Sigma-Aldrich, St. Louis, MO, USA), 20 mM HEPES (Sigma-Aldrich, St. Louis, MO, USA), 25 mM D-glucose (Sigma-Aldrich, St. Louis, MO, USA), 2 mM thiourea (Sigma-Aldrich, St. Louis, MO, USA), 5 mM Na-ascorbate (Sigma-Aldrich, St. Louis, MO, USA), 3 mM Na-pyruvate (Sigma-Aldrich, St. Louis, MO, USA), 2 mM CaCl_2_ (Sigma-Aldrich, St. Louis, MO, USA), 2 mM MgSO_4_ (Sigma-Aldrich, St. Louis, MO, USA)), saturated with carbogen with a controlled temperature (32–35 °C) for 15 min, after which the slices were incubated at room temperature of 23–25 °C. After 60 min of incubation, the sEPSC were recorded using the patch-clamp techniques (acute slices from outbreed albino-mice) or the field excitatory postsynaptic potentials were recorded (acute slices from C57BL/6J background mice) in standard ACSF (119 mМ NaCl, 2.5 mМ KCl, 1.25 mМ NaH_2_PO_4_, 24 mМ NaHCO_3_, 5 mМ HEPES, 12.5 mМ d-glucose, 2 mМ CaCl_2_, 2 mМ MgSO_4_). Patch electrodes were fabricated from borosilicate glass (2–3 MΩ), and filled with internal solution (120 mМ K-gluconate, 20 mМ KCl, 10 mМ HEPES, 0.2 mМ EGTA, 2 mМ MgCl_2_, 0.3 mМ Na_2_GTP, 2 mМ MgATP). 

In all voltage-clamp experiments, the neurons were held at −70 mV and sEPSC were recorded in presence of 1 µM TTX in internal solution. Only pyramidal neurons were included into statistics. They were identified by their shape and unique pattern of action potential generated using a step-protocol in current clamp mode. 

For extracellular field EPSP recordings, the 400 µm horizontal hippocampal slices were used, and the Schaffer collaterals were stimulated by a twisted bipolar electrode. fEPSPs were recorded in the CA1 stratum radiatum using a glass pipette containing ACSF (250–450 KΩ). fEPSPs were low-pass filtered at 400 Hz. 

For patch-clamp experiments with ChR_2_ opsin 7 AAV-injected mice were taken into experiments and for Opto-a1AR group 6 mice were injected and studied. Only one neuron per slice was patched, and if there were problems with recording after optogenetic stimulation slice was changed and never used again. For all fEPSPs experiments one slice for experimental and one slice for control group for each mouse were taken, so number of mice is equal to amount of slices (four mice in ChR2-expressed group and three in Opto- a1AR group). Also, one slice for recording was used and only one optogenetic stimulation was performed per slice.

Optogenetic light stimulation was performed by means of the blue LED (LED4D067, 470 nm, Thorlabs Inc., Newton, NJ, USA) with maximum intensity of 35 mW mm^−2^ with a maximum photo flux of 250 mW. 

## Data Availability

The presented data that is shown in this study is available by a request from the corresponding author.
